# A Machine Learning Approach to Predict Stress Hormones and Inflammatory Markers Using Illness Perception and Quality of Life in Breast Cancer Patients

**DOI:** 10.3390/curroncol28040275

**Published:** 2021-08-19

**Authors:** Irina Crumpei-Tanasă, Iulia Crumpei

**Affiliations:** 1 Department of Psychology, Faculty of Psychology and Educational Sciences, Alexandru Ioan Cuza University, 700554 Iași, Romania; 2 Faculty of Medicine, Grigore T. Popa University, 700115 Iași, Romania; iulia.g.crumpei@d.umfiasi.ro

**Keywords:** stress hormones, inflammatory markers, breast cancer, machine learning

## Abstract

Psychosocial factors have become central concepts in oncology research. However, their role in the prognosis of the disease is not yet well established. Studies on this subject report contradictory findings. We examine if illness perception and quality of life reports measured at baseline could predict the stress hormones and inflammatory markers in breast cancer survivors, one year later. We use statistics and machine learning methods to analyze our data and find the best prediction model. Patients with stage I to III breast cancer (N = 70) were assessed twice, at baseline and one year later, and completed scales assessing quality of life and illness perception. Blood and urine samples were obtained to measure stress hormones (cortisol and adrenocorticotropic hormone (ACTH) and inflammatory markers (c-reactive protein (CRP), erythrocyte sedimentation rate (ESR) and fibrinogen). Family quality of life is a strong predictor for ACTH. Women who perceive their illness as being more chronic at baseline have higher ESR and fibrinogen values one year later. The artificial intelligence (AI) data analysis yields the highest prediction score of 81.2% for the ACTH stress hormone, and 70% for the inflammatory marker ESR. A chronic timeline, illness control, health and family quality of life were important features associated with the best predictive results.

## 1. Introduction

Breast cancer is the most common malignancy in women [1]. In Romania, the Ministry of Health reports an increase of over 15% in breast cancer incidence for the past decades, with 9629 new cases in 2018 [2]. Moreover, Romania presents a worrisome increase in breast cancer mortality. While, between 2005 and 2012, the mortality had a descendent trend, since 2013, the number of deaths has been constantly rising. In Western Europe, the 5-year survival rate improved to 80% in the past decade. Romania has one of the lowest survival rates in the European Union, due to reduced breast cancer screening and delayed diagnosis [2,3].

The progress in oncology research improved survivorship in all cancer patients changing the focus from simply surviving to quality living after cancer. Resiliency grew into a central concept in cancer research as quality of life and distress became the sixth vital sign along with temperature, respiration, heart rate, blood pressure and pain [4,5,6,7]. The psychosocial factors turned out to be even more important during the Covid-19 pandemic when 64% of cancer patients (breast cancer patients included) experienced moderate or high stress associated with uncertainty, life changes, coping strategies, communication, experience or health services [8]. Generally, most cancer patients do no report clinical levels of depression [9], but the diagnosis, the symptoms and the treatment significantly decrease their reported quality of life [10].

The studies exploring the relationship between quality of life and cancer prognosis have produced contradictory results. While some investigations found that quality of life might be a prognostic factor for survival in cancer patients, in general [11,12], others report negative or inconsistent results [13]. Lee et al. [14] found that quality of life dimensions are not consistent predictors for illness outcome at first diagnosis. The association becomes significant in case of a relapse and is stronger later in the course of the recurrent disease [14]. The reason for the heterogeneous results remains unclear. The significant associations between quality of life and illness outcomes in more advanced forms of cancer might be linked to differences in illness perceptions.

The illness perceptions precede cancer diagnosis, but continue to develop and change after it. Generally, they are associated with cancer patients’ adherence to treatment, survival outcomes and perceived severity of symptoms [15,16,17]. In particular, breast cancer patients report more negative illness perceptions [18]. The breast cancer patients who report more negative emotions associated with cancer and expected more negative consequences related to their illness have higher mortality rates [15] and poorer health related quality of life [19]. The link between illness perception and mortality rates in cancer patients could be explained through the body’s stress response.

Multiple studies report dysregulations in endocrine and sympathetic nervous systems in breast cancer patients [20], and stressful life events have been associated with physiologic disturbances. For example, acute stress elicits an adaptive response in the human body, stimulating the nervous and the endocrine systems to cope with the stressor. Nonessential functions of the body such as reproduction, digestion and growth are inhibited. Glucose and free fatty acids are increased. Stress hormones as adrenaline and cortisol are released to prepare the person to fight the threat. The hypothalamic–pituitary–adrenal (HPA) axis is one system responsible for the body’s stress response. In stressful situations, the paraventricular nucleus in the hypothalamus discharges corticotropin–releasing hormone (CRH), causing the pituitary to release adrenocorticotropic hormone (ACTH), which, in turn, will determine the release of cortisol in the adrenal glands. To avoid the system overuse, the cortisol then obstructs the discharge of CRH. In short-term, these reactions are helpful and necessary. Generally, the body returns to its normal functions once the perceived danger has passed [21]. However, breast cancer is a more chronic stressor, and patients might feel vulnerable for longer periods of time. In chronic stress, the production of stress hormones loses its balance and the body cannot return to normal [22]. Several studies show that prolonged stressful experiences are associated with both hyper- and hypo-cortisol regulation [23]. The cortisol has a strong anti-inflammatory function, preventing widespread tissue and nerve impairment due to inflammation [24], but long-lasting chronic stress results in cortisol dysfunction associated with an unmodulated inflammatory response to both pathogens and psychological stress [25]. These dysfunctions could explain the way how the psychosocial factors influence cancer prognosis and survival outcomes [26].

Despite the consistent body of research showing a significant relationship between psychosocial factors and breast cancer survival [27], the physiological mechanisms involved are still controversial. While several studies suggest that the HPA axis is an important biological system associated with psychosocial factors and survival outcomes [28,29], others find no significant relationships between stress markers and psychological measures [30,31].

In the present study, we examine whether psychosocial factors (illness perception and quality of life reports) can predict stress hormones and inflammatory markers in breast cancer survivors, one year later. We conjecture that lower levels of quality of life at baseline yield higher levels of stress hormones and inflammatory markers one year later. Based on previous findings, we expect that negative illness perceptions predict higher levels of stress hormones and inflammatory markers over time. We use statistics and machine learning methods to analyze our data and build a best prediction model.

## 2. Materials and Methods

### 2.1. Participants

The patients were recruited in one medical establishment in Iasi, where they came for their periodic medical examination. Baseline data collection took place during March–May of 2018. All participants were invited to also take part in the second assessment, one year apart from the first, when they were scheduled for their next check-up. The inclusion criteria for all participants comprised a diagnosis of stage I to III breast cancer and treatment completion. The exclusion criteria were potentially fatal comorbid diagnosis, a stage IV cancer diagnosis.

### 2.2. Measures

The Quality of Life Index (QLI)–Cancer III Version [32], was used to measure both satisfaction and importance regarding different aspects of life. Final scores report satisfaction with the aspects of life valued by the person. It contains 4 sub-scales that offer independent scores measuring satisfaction on different domains: health and functioning (α = 0.80), psychological/spiritual (α = 0.84), social and economic (α = 0.73) and family (α = 0.75). Items can be summed up to generate a total quality of life score (α = 0.90).

The revised version of the Illness Perception Questionnaire (IPQ-R) [33] was used to assess illness perception. The questionnaire measures nine dimensions of illness perception. Five dimensions assess negative illness perceptions such as attributing more negative consequences, emotions and symptoms to the illness and perceiving it as chronical: identity, timeline, consequences, time cyclical and emotional representations. Higher scores on these dimensions indicate a more negative illness perception. The other three dimensions assess positive perceptions as treatment control, personal control and illness coherence, with higher scores indicating more positive beliefs. The questionnaire was used to measure illness perceptions among patients with different diseases, including cancer with good psychometric properties [34,35,36]. The Cronbach’s alpha coefficients for the translated Romanian version ranged between 0.68 and 0.85 for the 9 dimensions.

The blood and urine samples were obtained at baseline, and one year later, to measure stress hormones (cortisol and ACTH) and inflammatory markers (c-reactive protein (CRP), erythrocyte sedimentation rate (ESR) and fibrinogen). The samples were processed in the hospital laboratory. We chose these markers based on previous studies identifying stress hormones and inflammatory markers associated with illness evolution in cancer patients and on the laboratory tests routinely available in the medical institution. The participants were instructed to collect their urine over a period of 24 h. They were asked to urinate at 7 o’clock in the morning and to throw away the urine. For the next 24 h, they were told to collect all urine discharges in a clean 2–3 L container, until 7 am the next day. They were asked to homogenize the collected urine by stirring, measure the entire quantity and retain 10 mL in a disposable plastic container. The samples were to be stored at 2–8 °C until they were effectively processed. We clearly explained the procedure and the importance of collecting all urine discharges over the day. The patients knew they would receive the test results and discuss them with their doctor. Noncompliance with the sampling instructions should be minimal [37]. The urine was used to test levels of free urinary cortisol. The blood samples were drawn between 8.00 and 11.30 am for each patient and for both assessments; the patients were instructed to fast after midnight and drink liquids as needed.

### 2.3. Procedure

After the study was approved by the review board, we approached prospective participants and explained the objectives, risks and benefits of our study. The participants were informed that they were free to withdraw at any time. The study discussions took place away from any member of the patient’s medical team to ensure that they would not feel any outside constraint to participate. After we obtained their written consent, they received the self-report questionnaires. Quality of life and illness perception were measured only at baseline. The blood and urine samples were obtained as part of their periodical check-ups. One year later, they repeated the biological tests.

### 2.4. Data Analysis

The SPSS 25.0 program (IBM Corp, Armonk, NY, USA) was used for preliminary data analysis. We used descriptive statistics, including frequencies, percentage, means and standard deviation to describe our sample at baseline. The Pearson correlations were used to explore the relationships between the research variables. Multiple hierarchical regression was used to predict total quality of life using illness perception domains. ACTH was also predicted using family quality of life. Paired samples T tests were used to compare initial levels of stress hormones and inflammatory markers with values obtained one year later. There were 3% missing data, which were replaced with the sample mean.

A priori power analysis was performed to estimate the minimal number of patients needed for hierarchical linear regression. Power calculations were performed with G*Power 3.1 (Franz Faul, Kiel University, Germany) for a power level of 0.80 and 5% level of significance, and the sample size was estimated at 61 participants. Given a 10% probability of loss of participants and for a higher accuracy, we addressed more patients than the minimal calculated.

For more in-depth analysis, we used machine learning to explore the predictive value of the chosen variables. We tested six different algorithms on our datasets: logistic regression, linear discriminatory analysis, K-nearest neighbors classification and regression trees, Naive Bayes, and support vector machine. We chose these machine learning algorithms based on previous research studying breast cancer risk calculation and prognosis using machine learning. We also used the support vector machine algorithm as multiple studies report that this algorithm was the most accurate in predicting breast cancer risk.

## 3. Results

### 3.1. Data Analysis Using SPSS

#### 3.1.1. Characteristics of Breast Cancer Patients

A total of 125 breast cancer patients were assessed for eligibility; 81 agreed to take part in our study and completed a baseline assessment; 11 women of the original sample did not take part in the second assessment, one year later. The analytic sample therefore included 70 breast cancer patients, resulting in an 86% retention rate. No significant differences existed in the baseline data (of age and explored variables) of the participants who took part in the second assessment and those who dropped out. The mean age of the participants was 53 years (SD = 11.6). The mean duration between completion of cancer treatments and study entry was 4.7 years (SD = 5.01) (Table 1).

#### 3.1.2. Quality of Life and Illness Perception

We conducted Pearson correlations to explore the associations between the quality of life and illness perception dimensions (Table 2).

Our findings are that the women who feel their illness is more permanent manifest a lower level of psychological quality of life. A cyclical perception of symptoms is associated with lower health-related quality of life. Patients who associate more negative consequences to the illness show lower levels of health, social and psychological quality of life. Perceiving higher coherence in one’s symptoms and associating fewer negative emotions to the illness is associated with higher levels of quality of life in all domains. The sociodemographic and illness-related variables were examined in relation to illness perception and quality of life. The older women reported perceiving less illness coherence (r = −0.28, *p* = 0.019). Women who are closer to the time of their treatment and diagnosis associate more negative emotions with their illness (r = −0.34, *p* = 0.005). There are no other significant correlations between age, time since treatment, number of births and illness perception or quality of life.

We conducted Mann–Whitney tests to examine the differences between women who had mastectomy and those with conservative interventions. Women with conservative intervention perceived more personal control over their illness (M = 42.08) compared with women who had mastectomy (M = 30.56, U = 308.50, *p* = 0.026). There are no other differences between the two groups’ quality of life and illness perception.

We ran multiple regression analysis to explore whether illness perception dimensions predict quality of life. We selected dimensions that showed significant correlations to the total quality of life score. Our predictors were: time cyclical, consequences, coherence and emotions. The results of the regression indicate that the model explained 42% in the variance. It was found that illness coherence and emotion representations significantly predicted global quality of life (Table 3).

#### 3.1.3. Stress Hormones and Psychosocial Factors

**Figure 1:**
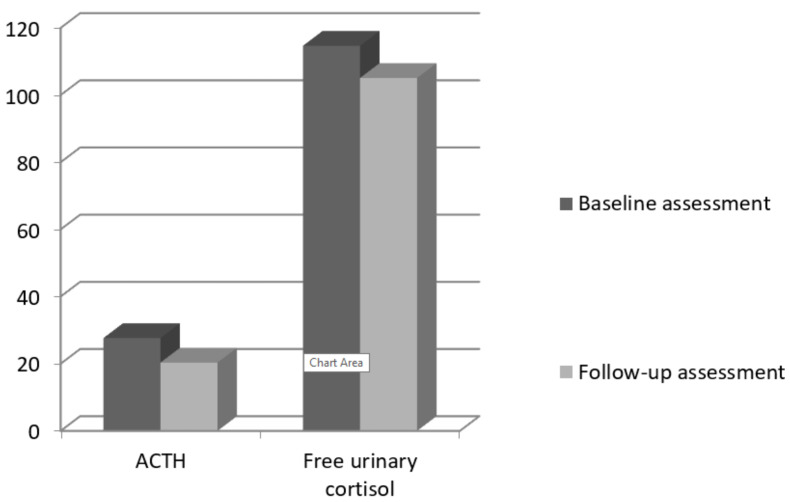
Baseline and follow-up means for ACTH and cortisol.

To explore the changes in stress hormones over 12 months, we computed paired samples *t*-tests between the two assessments. There were no significant differences between the two measures for ACTH: t (69) = 1.45, *p* = 0.150 or for cortisol: t (69) = 0.99, *p* = 0.325 (Figure 1, Table 4).

We also conducted Pearson correlations between quality of life and illness perception dimensions and stress hormones (Table 5). Women with higher quality of life in their family have lower levels of ACTH, one year later (r = −0.57, *p* < 0.001). There is also a marginal significant correlation between treatment control and ACTH. Women who perceive having more control over their treatment exhibit lower levels of ACTH, one year later (r = −0.24, *p* = 0.090). There are no significant correlations between free urinary cortisol at follow up and psychosocial factors.

We used hierarchical multiple regression analysis to explore if familial quality of life and perception of treatment control at baseline predict ACTH levels, one year later. We controlled for age, cancer stage and years since the diagnosis. The regression results indicate that the model explained 48% in the variance of the variable. It was found that family quality of life significantly predicted ACTH levels, one year later (Table 6).

#### 3.1.4. Inflammatory Markers and Psychosocial Factors

To explore the changes in the inflammatory markers over 12 months, we computed paired samples *t*-tests between the two assessments. There were no significant differences between the two measures for ESR: t (69) = 1.45, *p* = 0.151 or CRP: t (69) = 0.81, *p* = 0.41. Fibrinogen at the second assessment was significantly lower (M = 359.64), compared with the baseline assessment (M = 380.98), t (69) = 3.24, *p* = 0.002.

We conducted Pearson correlations between quality of life, illness perception dimensions and levels of inflammatory markers. Women who perceive their illness as being more chronic at baseline have higher levels of ESR (r = 0.34, *p* = 0.015) and fibrinogen (r = 0.26, *p* = 0.061), one year later. There are no other significant correlations between inflammatory markers and psychosocial factors (Table 7).

### 3.2. Data Analysis with Artificial Intelligence (AI) Methods

We created five .csv files using the general database (Table 8. We placed the eight illness perception features and the four quality of life features in columns. The last column contained the target variables, the stress hormones: ACTH, CLU and the inflammatory markers CRP, ESR and FBG, as indicated in the image below. At the same time, all missing values were replaced with the average score for each variable.

We assessed multiple different machine learning algorithms on the 5 datasets in Python (Python Software Foundation. Python Language Reference, version 2.7. Available at http://www.python.org) with scikit-learn. We used the same test harness to evaluate the algorithms, and we summarized the results both numerically and using a box and whisker plot. We used the gradient boosting ensemble from scikit-learn for classification and then explored the effect of the gradient boosting model hyperparameters on the model performance.

We used feature selection for preparing machine learning data in Python with scikit-learn and applied 4 different automatic feature selection techniques on our datasets: univariate selection, recursive feature elimination, principal component analysis and feature importance. Appendix A contains more details about the process of comparing the machine learning algorithms in Python with scikit learn.

#### 3.2.1. Comparing Consistently the Machine Learning Algorithms

We evaluated each algorithm identically on the same data, on a consistent test chain. We compared six different algorithms: logistic regression (LR), linear discriminatory analysis (LDA), K-nearest neighbors (KNN) classification, regression trees (CART), Naive Bayes (NB) and support vector machine (SVM) [38].

We analyzed a standard binary classification dataset (ACTH.csv), with two classes and twelve numeric input variables at different scales. The 10-fold cross-validation procedure was used to evaluate each algorithm, configured with the same random seed to ensure that the same divisions were performed with the training data and that each algorithm was evaluated in exactly the same way (Appendix B) (Table 9).

Our results suggest that both KNN (k nearest neighbors) and SVM (support vector machine) are algorithms worthy of further study in connection with this problem.

#### 3.2.2. Gradient Boosting for Classification

We analyzed the use of gradient boosting for a classification problem. We included a more detailed description of this process in Appendix C [39,40,41,42]. We loaded the ACTH.csv dataset and evaluated a gradient boosting algorithm on this dataset. We assessed the model using repeated stratified k-fold cross-validation, with three repetitions and 10 folds. We reported the mean and standard deviation of the model accuracy for all iterations and folds (Appendix D).

Running the example, we obtained a mean accuracy of 0.695 and a standard deviation of 0.142 for this model.

##### Grid Search for Hyperparameters

We used a search process to find model hyperparameters that work well or best for a given predictive modeling problem. Popular search processes include a random search and a grid search.

We analyzed the usual grid search intervals for the key hyperparameters of the gradient growth algorithm that we could use as a starting point for our own projects. This was done using the GridSearchCV class and specifying a dictionary that maps the name of the model hyperparameters to the searchable values.

In this case, we looked in the grid for four key hyperparameters for gradient boosting: the number of trees used in the ensemble, the learning rate, the size of the sub-sample used to train each tree and the maximum depth of each tree. We used for each hyperparameter a series of values widely used for the good performances they achieve. Each configuration combination was evaluated using repeated k-fold cross-validation, and the configurations were compared using the average score, in this case, the accuracy of the classification. The complete example of grid search of the key hyperparameters of the gradient growth algorithm in our classification dataset is listed in Appendix E. The configuration with the best score is reported first, followed by the scores for all other configurations considered.

We observed that a configuration with a learning rate of 0.0001, maximum depth of 3 levels, 10 trees and a sub-sample of 50% performed best with a classification accuracy of about 81.2%. The model could work better with multiple trees, such as 1000 or 5000; these configurations were not tested to ensure that the grid search is completed within reasonable time.

The example in the appendix demonstrates this on our binary classification dataset (Appendix F). The example fitted the model of the gradient boosting assembly on the entire dataset and was then used to make a prediction on a new dataset, as we would do in applications.

#### 3.2.3. Feature Selection for Machine Learning in Python

The features of the data used to train machine learning models have significant influence on the performance that can be achieved. Irrelevant or partially relevant features may have a negative impact on the model’s performance. In the following, we present the automatic feature selection techniques we used to prepare the Python machine learning data with scikit-learn [43,44].

##### Feature Selection

Feature selection is a process in which we select the features from our data that contribute most to the prediction variable or the output in which we are interested. Irrelevant features in data can reduce the accuracy of many of the models, especially in the case of linear algorithms, such as linear and logistic regression.

##### Feature Selection for Machine Learning

We list here the 4 recipes for selecting the features for machine learning in Python, which we used on our database. Each recipe was designed to be complete and independent so that we can copy and paste it directly into the project and use it immediately. The recipes use our datasets to demonstrate how to select features. This is a binary classification problem in which all attributes are numeric.

##### Univariate Selection

Statistical tests can be used to select those characteristics that have the strongest relationship with the output variable. The scikit-learn library offers the SelectKBest class that can be used with a suite of different statistical tests to select a specific number of features. Many different statistical scans can be used with this selection method. For example, the F-value ANOVA method is suitable for numeric inputs and categorical data. It can be used via the *f_classif ()* function. We selected the best 4 features using this method in the example below; see Appendix G. The features with indices 0 (IPQtimeline), 3 (IPQperscontrol), 8 (hfsub) and 11 (famsub) had the highest scores.

##### Recursive Feature Elimination

The recursive feature elimination (or RFE) works by recursively deleting features and building a model on those remaining attributes. We used the accuracy of the model to identify which attributes (and combination of attributes) contribute the most to predicting the target variable.

The example below uses RFE on the logistic regression algorithm to select the first 3 features. The chosen algorithm is not too important, as long as it is skillful and consistent (Appendix H). RFE chose the first 3 features as IPQtimeline, hfsub and pspsub.

##### Principal Component Analysis

The Principal Component Analysis (or PCA) uses linear algebra to transform a dataset into a compressed form. A feature of PCA is that we can choose the number of dimensions or main components in the transformed result. In our example, we used PCA and selected 3 main components (Appendix I) so that the transformed dataset does not resemble the source data.

##### Feature Importance

Bagged decision trees, such as random forest and extra trees, can be used to estimate feature importance.

For the example in Appendix J, we built an *ExtraTreesClassifier* for datasets (Appendix J). We assign an importance score to each attribute; the higher the score, the more important the attribute (e.g., IPQtimeline, IPQtimecycle and famsub).

Thus, feature selection prior to entering the data in the model lead to reduced overfitting, improved accuracy and reduced training time.

#### 3.2.4. Machine Learning Results

**Figure 2:**
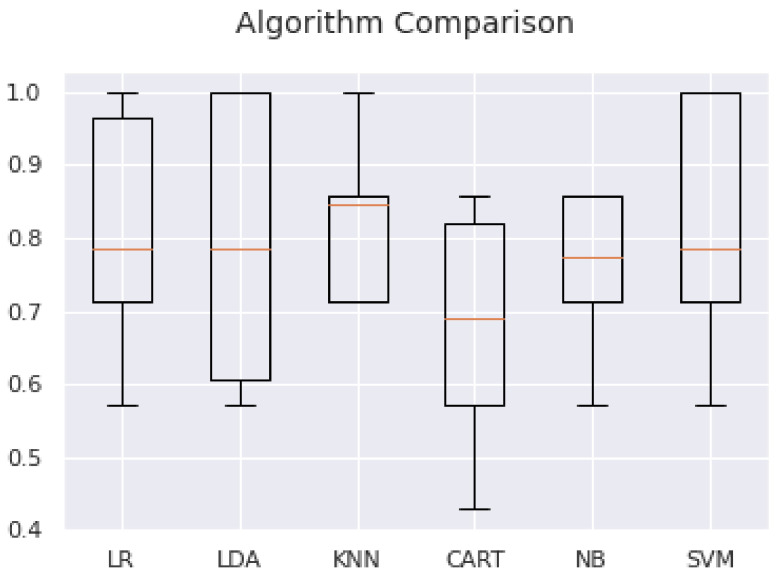
Comparison of machine learning algorithms for the feature reduction analysis.

For ACTH, the use of features 0, 1, 3, 8, 10 and 11 (selected as important through the reduction methods) in the algorithms SVM: 0.809524 (0.132993) or KNN: 0.778571 (0.157952) lead to the best results. We ran the test program for machine learning algorithms on ACTH.csv, which contains all the features, and on ACTH_1.csv, which contains only the features 0, 3, 8 and 11. The results are collected in Table 10 (Figure 2):

As expected, there is an improvement in all variables.

The GBM algorithm improved from mean accuracy: 0.695 (0.142) to 0.811905 using {‘learning_rate’: 0.0001, ‘max_depth’: 3,’n_estimators’: 10, ‘subsample’: 0.5} and the grid search method.

For CLU, using features 2, 3, 8, 9, 10 and 11 (selected as important through the reduction methods) introduced in algorithms CART: 0.523810 (0.208656) or NB: 0.461905 (0.149147) leads to the best results. The GBM algorithm improved from mean accuracy: 0.459 (0.207) to 0.521429 using {‘learning_rate’: 0.0001, ‘max_depth’: 3, ‘n_estimators’: 10, ‘subsample’: 0.5} and the grid search method.

For CRP, using features 0, 1, 3, 5, 9 and 10 (selected as important through the reduction methods) introduced in algorithms LR: 0.626190 (0.142081) or LDA: 0.590476 (0.219461) leads to the best results. The GBM algorithm improved from mean accuracy: 0.560 (0.147) to 0.616667 using {‘learning_rate’: 1.0, ‘max_depth’: 9, ‘n_estimators’: 10, ‘subsample’: 0.7} and the grid search method.

For ESR, using features 0, 4, 5, 7, 8 and 11 (selected as important through the reduction methods) introduced in algorithms NB: 0.664286 (0.234847) or SVM: 0.664286 (0.163039) leads to best results. The GBM algorithm improved from mean accuracy: 0.594 (0.141) to 0.700000 using {‘learning_rate’: 0.01, ‘max_depth’: 3, ‘n_estimators’: 100, ‘subsample’: 0.5} and the grid search method.

For FBG, using features 3, 4, 5, 7, 8 and 10 (selected as important through the reduction methods) introduced in algorithms NB: 0.628571 (0.232115) or SVM: 0.680952 (0.085317) leads to best results. The GBM algorithm improved from mean accuracy: 0.552 (0.176) to 0.680952 using {‘learning_rate’: 0.0001, ‘max_depth’: 3, ‘n_estimators’: 10, ‘subsample’: 0.5} and the grid search method.

Initial results for all variables are collected in Table 11.

Completing the analysis with artificial intelligence (AI) methods, the highest prediction score was obtained for a GBM algorithm after adjusting the hyperparameters, 81.2% for the ACTH stress hormone and 70% for the inflammatory marker ESR. Selecting the relevant features prior to entering the data in the model, we obtained better results for all 7 machine learning algorithms used, as expected.

## 4. Discussion

### 4.1. Illness Perception and Quality of Life

Consistent with previous research, our results suggest that negative illness perceptions are associated with lower quality of life in breast cancer patients [19,45]. The patients who perceive breast cancer as a serious condition, with major consequences on their lives, show lower levels of health, social and psychological quality of life. Moreover, seeing cancer as a permanent condition, with an unpredictable course, is associated with lower psychological and consequentially lower health quality of life. Previous studies found similar results, especially in older breast cancer patients reporting less positive illness perception and lower wellbeing [34]. Our findings also underline the predictive value of patients’ illness perception. Emotion representations and illness coherence significantly predicted global quality of life. Our results are consistent with the studies showing that more negative illness perception is associated with lower wellbeing and quality of life [19,34]. Emotional representations were the strongest predictor. While emotional representations are reported as an important quality of life predictor in other studies as well, illness coherence is less central [19,34]. The Romanian patients might have lower cancer literacy [46], which could explain the stronger relationship between illness coherence and quality of life. Those who better understood their illness and symptoms reported higher quality of life in all domains. The older women reported feeling more puzzled about their symptoms, showing less illness coherence [34]. The intervention efforts should take into account this specific need, giving patients more information about breast cancer symptoms and signs, helping them to perceive cancer as more coherent.

### 4.2. Ilness Perception, Quality of Life and Stress Hormones

The present study investigated if illness perception and quality of life can predict the levels of stress hormones one year later. Traditional regression analysis shows that ACTH levels could be predicted using family quality of life. Women who report higher quality of life in their family have lower levels of ACTH one year later. We could not find other associations between psychosocial measures and ACTH. This is consistent with previous research on the subject reporting inconclusive results [22]. The importance of family quality of life can be explained by the extensively documented role of perceived social support in breast cancer patients’ adjustment [34], and good family life may have a protective role over time.

The artificial intelligence analysis yielded the highest prediction score for ACTH, 81.2%. The most important features, selected through the reduction methods, were perception of a chronic timeline, perceived personal control, health and family quality of life. The importance of the perceived personal control and the chronic timeline were also underlined in a study exploring breast cancer patients’ perceptions of gene expression profiling [47]. The perceived personal control and the will to prevent a chronic timeline were part of the patients’ tendency to overestimate the importance of gene expression profiling. Perceived helplessness and the fear of the chronic timeline could lead to stress, explaining higher levels of ACTH. Illness perceptions and quality of life are associated with cancer mortality risk [15], and our findings highlight parts of the biological mechanism involved.

We did not find any significant Pearson correlation between the free urinary cortisol at follow-up and the psychosocial factors. The learning machine algorithm found a weak prediction score of 52% for urinary cortisol using perceived illness consequences, social, psychological and family quality of life as predictors, which is close to the random guess and validates the above statistical analysis. Breast cancer is accompanied by long-lasting stress, which is known to result in the cortisol dysfunction associated with an unmodulated inflammatory response [25]. While some studies have found positive relationships between cortisol and psychosocial factors in cancer patients, others have shown no relationship [48,49].

### 4.3. Quality of Life, Illness Perception and Inflammatory Markers

We also explored the relationship between quality of life, illness perceptions and inflammatory markers. Most psychosocial measures do not correlate with inflammatory markers. However, women who perceive their illness as being more chronic at baseline have higher levels of ESR and fibrinogen, one year later. The machine learning algorithm found a 70% prediction score for ESR (using the perceived illness coherence and identity, chronic timeline and treatment control as predictors), and a 68% prediction score for fibrinogen. The most important features were the illness coherence and identity, health and psychological quality of life. For CRP, the algorithm found a 61% prediction score, based on perceived chronic timeline, illness coherence, social and psychological quality of life. Previous studies also suggest that negative perceptions on consequences, timeline, identity and emotions are associated with higher mortality risks [15]. Inflammation might be the frame explaining how illness perception can predict breast cancer survival outcomes. Previous studies show that breast cancer survivors have high CRP levels immediately after treatment, but they tend to normalize with the passage of time [50]. These patients were in various moments of post-treatment time, and this high variability might explain the lower prediction scores. The mean duration between completion of cancer treatments and study entry was 4.7 years.

## 5. Limitations and Future Directions

The results should be considered within the limitations of this study. Our patients filled in the self-report measures when they came for their periodical medical examinations. Their answers might have been influenced by the stressful situation. As such, it would be important for future research to examine psychosocial factors in different contexts, using multiple recurrent assessments.

Both stress hormones and inflammatory markers oscillate from the time of diagnosis through treatment and survival. Our sample consisted of breast cancer survivors with varying years since diagnosis. Future studies should try to have more homogenous samples in terms of time passed since diagnosis and treatment completion. Larger samples would also offer more reliable results.

We could only measure psychosocial factors at baseline. It would have been useful to have a second assessment one year later. The intermediate measures of biological markers could help to better understand the dynamics of their evolution.

## 6. Conclusions

Our study adds to the growing body of research exploring the relationship between psychosocial factors and biological markers in cancer patients. For many years, unidentified psychosocial distress has been linked to weaker adherence to treatment recommendations, more healthcare needs for nonmedical concerns, maladaptive coping mechanisms and chronic mental health issues in cancer patients and survivors [51]. Three conclusions can be drawn from this study. First, perceived illness coherence and negative emotions are significant predictors of breast cancer patients’ quality of life. While, in previous studies, negative emotions were strongly associated with quality of life, the predictive value of illness coherence may be more specific to the Romanian context, where cancer literacy is lower. Older patients report lower illness coherence. Psychosocial intervention efforts should include illness coherence among their objectives, prioritizing older patients. Second, perception of a chronic timeline, perceived personal control, health and family quality of life at baseline show an 80% prediction score for ACTH, one year later. Familial support through cancer survivorship might be a vital resource. Addressing strained family relations and increasing personal control through counselling and psychotherapy could help cancer prognosis. Third, perceived illness coherence and identity, chronic timeline and treatment control at baseline show a 70% prediction score for ESR. Stress hormones and inflammation processes might be the frame explaining how illness perception and quality of life can predict breast cancer survival outcomes.

## Figures and Tables

**Table 1:** Demographic data.

	Mean (SD)	N	%
Age (mean)	53(11.6)		
**Years since treatment**	4.7(5.01)		
6 months–1 year		23	32.8
1–5 years		22	31.4
5–10 years		13	18.5
10–20 years		12	17.1
**Type of intervention**			
Conservative intervention		22	31
Mastectomy		48	69
**Type of treatment**			
Radiotherapy		11	15.7
Chemotherapy		16	22.8
Radio + Chemotherapy		38	54.2
Other treatments		5	7.14
**Relationship status**			
Married/in a relationship		50	71.4
Single/divorced		20	28.5
**Education level**			
At least high school		63	90
**Cancer stage**			
Stage I		27	38.5
Stage II		30	42.8
Stage III		13	18.5

**Table 2:** Correlations between quality of life and illness perception dimensions.

	Health	Social	Psychological	Family
Timeline	−0.17	−0.21	−0.30 *	−0.14
Time cyclical	−0.25 *	0.22	−0.15	−0.17
Consequences	−0.44 **	−0.36 **	−0.38 **	−0.20
Personal control	−0.01	0.08	0.006	−0.02
Treatment control	0.16	0.15	0.26 *	0.16
Coherence	0.40 **	0.33 **	0.36 **	0.27 *
Emotions	−0.51 **	−0.54 **	−58 **	−0.35 **
Identity	0.08	0.04	0.05	−0.09

Note: * = *p* < 0.05, ** = *p* < 0.001. N = 70.

**Table 3:** The hierarchical regression analysis for the illness perception dimensions predicting total quality of life.

Variables	Quality of Life		
	ΔR^2^	ΔF	Β
Time cyclical	0.06	4.11 *	−0.02
Consequences	0.14	11.57 ***	−0.19
Coherence	0.11	9.93 **	0.22*
Emotions	0.10	11.36 ***	−0.40 ***

Note: ΔR^2^: R square change, ΔF: F change * = *p* < 0.05, ** = *p* < 0.01 *** *p* < 0.001. N = 70 R^2^ = 0.42, F = 11.19 ***.

**Table 4:** Means and *t*-tests for the biological markers.

	First Assessment		Second Assessment		*t*-Test
	*M*	*SD*	*M*	*SD*	
ESR (mm/1 h)	6.63	5.208	5.04	2.87	1.45
Fibrinogen (mg/dL)	385.53	62.81	360.38	61.200	3.24 **
CRP (mg/dL)	0.67	2.26	0.17	1.37	0.81
ACTH (pg/mL)	26.76	26.01	20.34	32.29	1.45
Cortisol	121	56.83	105.09	57.26	0.99

** *p* < 0.01.

**Table 5:** Correlations between quality of life, illness perception dimensions and stress hormones.

	qolH	qolS	qolP	qolF	ipT	ipCy	ipCo	ipP	ipT	Ipcoh	ipE	ipI
Cortisol	0.09	−0.04	−0.06	0.007	0.09	−0.12	−0.22	0.06	−0.08	0.06	0.01	−0.21
ACTH	−0.13	−0.12	−0.06	−0.57 **	0.08	−0.01	−0.04	−0.11	−0.24 ^	−0.06	0.05	0.06

Note: ^ = *p* < 0.10, ** = *p* < 0.001. N = 70.

**Table 6:** The hierarchical regression analysis for the psychosocial factors predicting ACTH.

Variables	ACTH			
	ΔR^2^	ΔF	Β	Β
			Step 1	Step 2
Step 1	0.02	0.16		
Age			0.07	0.09
Years since diagnosis			−0.12	−0.09
Cancer stage			−0.02	−0.04
Step 2	0.48	10.13 ***		
Family quality of life				−0.66 ***
Treatment control				−0.07

Note: ΔR^2:^ R square change, ΔF: F change ** *p* < 0.001. N = 70 ACTH Follow-up Step 1: R^2^ = 0.02, F = 0.16 Step 2: R^2^ = 0.38, F = 4.23 ***.

**Table 7:** Correlations between quality of life, illness perception dimensions and inflammatory markers.

	qolH	qolS	qolP	qolF	ipT	ipCy	ipCo	ipP	ipT	ipcoh	ipE	ipI
ESR	−0.18	−0.10	−0.13	−0.08	0.34 *	0.07	0.08	−0.06	0.06	0.01	0.23	−0.06
CRP	−0.21	−0.02	0.07	−0.04	−0.08	0.12	−0.07	0.04	0.13	−0.03	−0.01	−0.25
FBG	0.09	0.03	0.10	−0.05	0.26 ^	−0.02	−0.06	0.09	0.09	0.03	0.02	−0.01

Note: *^ = p* < 0.10, * = *p* < 0.05. N = 70.

**Table 8:** Data bases, targets and features.

Data Bases	Targets = 0 (Decrease or Stagnation) = 1 (growth)		Features	
	Stress Hormones	Inflammatory markers	Illness perception	Quality of life
ACTH.csv	ACTH	CRP	0 IPQtimeline (−)	8 hfsub (health QL)
CLU.csv	CLU	ESR	1 IPQticyclical (−)	9 socsub (social QL)
CRP.csv		FBG	2 IPQconsequences (−)	10 pspsub (psychological QL)
ESR.csv			3 IPQpersonal control (+)	11 famsub (family QL)
FBG.csv			4 IPQtreatment control (+)	
			5 IPQillness coherence (+)	
			6 IPQemotional (−)	
			7 IPQidentity (−)	

**Table 9:** Algorithm comparison.

	LR	LDA	KNN	CART	NB	SVM
M (SD)	0.738 (0.109)	0.771 (0.171)	0.778 (0.157)	0.623 (0.128)	0.740 (0.188)	0.809 (0.132)

**Table 10:** ACTH: results of machine learning algorithms for all features and for the feature reduction situation with only relevant features.

Selected Features	All Features
LR: 0.800000 (0.159079)	LR: 0.754762 (0.180089)
LDA: 0.800000 (0.182946)	LDA: 0.752381 (0.213809)
KNN: 0.811905 (0.090633)	KNN: 0.795238 (0.100000)
CART: 0.680952 (0.139728)	CART: 0.638095 (0.203373)
NB: 0.769048 (0.092857)	NB: 0.695238 (0.118952)
SVM: 0.814286 (0.169633)	SVM: 0.811905 (0.127975)

**Table 11:** Results of the analysis of databases with 7 machine learning algorithms, part of AI.

	LR	LDA	KNN	CART	NB	SVM	GBM
ACTH	0.738095	0.771429	0.778571	0.623810	0.740476	0.809524	0.811905
CLU	0.385714	0.304762	0.390476	0.523810	0.461905	0.376190	0.521429
CRP	0.626190	0.590476	0.404762	0.452381	0.550000	0.364286	0.616667
ESR	0.578571	0.535714	0.466667	0.566667	0.664286	0.664286	0.700000
FBG	0.552381	0.609524	0.595238	0.464286	0.628571	0.680952	0.680952

## Data Availability

The data presented in this study are available upon request from the corresponding author.

## Appendix A

### Appendix A.1. Comparing the Machine Learning Algorithms in Python with Scikit-Learn

We consistently compared the performance of several machine learning algorithms. In the following, we describe how we created a test chain to compare several machine learning algorithms in Python with scikit-learn. We used this test chain as a template for machine learning problems in the analyzed database, and we add several different algorithms to compare them [38].

### Appendix A.2. Choosing the Best Machine Learning Model

There are several good models for machine learning projects. Each model has different performance features. Using sampling methods, such as cross-validation, one gets an estimate of how exactly each model can predict unseen data. We use these estimates to choose one or two of the best models from the suite of models.

### Appendix A.3. Careful Comparison of Machine Learning Models

When working with a new dataset, it is recommended to visualize the data using different techniques and observe them from different perspectives. The same idea applies to model selection. We used a number of different ways to look at the estimated accuracy of machine learning algorithms to choose one or two to complete the analysis. One can use different visualization methods to highlight the average accuracy, the variance and other properties of the precisions’ distribution model. We continued by undertaking this in scikit-learn from Python.

## Appendix B

# compare algorithms
import pandas
import matplotlib.pyplot as plt
from sklearn import model_selection
from sklearn.linear_model import LogisticRegression
from sklearn.tree import DecisionTreeClassifier
from sklearn.neighbors import KNeighborsClassifier
from sklearn.discriminant_analysis import LinearDiscriminantAnalysis
from sklearn.naive_bayes import GaussianNB
from sklearn.svm import SVC

# load dataset
dataframe = pandas.read_csv(‘ACTH.csv’)
array = dataframe.values
X = array[:,0:12]
Y = array[:,12]

# prepare models
models = []
models.append((‘LR’, LogisticRegression()))
models.append((‘LDA’, LinearDiscriminantAnalysis()))
models.append((‘KNN’, KNeighborsClassifier()))
models.append((‘CART’, DecisionTreeClassifier()))
models.append((‘NB’, GaussianNB()))
models.append((‘SVM’, SVC()))
# evaluate each model in turn
results = []
names = []
scoring = ‘accuracy’
for name, model in models:
kfold = model_selection.KFold(n_splits = 10, shuffle = True)
cv_results = model_selection.cross_val_score(model, X, Y, cv = kfold, scoring = scoring)
results.append(cv_results)
names.append(name)
msg = “%s: %f (%f)” % (name, cv_results.mean(), cv_results.std())
print(msg)

# boxplot algorithm comparison
fig = plt.figure()
fig.suptitle(‘Algorithm Comparison’)
ax = fig.add_subplot(111)
plt.boxplot(results)
ax.set_xticklabels(names)
plt.show()

## Appendix C

### Appendix C.1. Developing a Gradient Boosting Machine Ensemble in Python

Gradient boosting machine is a powerful ensemble machine learning algorithm that uses decision trees. Boosting/stimulation is a general ensemble technique that involves the sequential addition of models to the ensemble, where subsequent models correct the performance of previous models [39,40]. AdaBoost was the first algorithm to fulfill its promise of boosting. Increasing the gradient is a generalization of AdaBoosting, improving the performance of the method and introducing ideas from the bootstrap aggregation to further improve the models, such as random sampling of samples and features when fitting assembly members. Gradient amplification works well, if not best, on a wide range of tabular datasets, and versions of the algorithm such as XGBoost and LightBoost often play an important role in winning international machine learning competitions, such as Kaggle [41].

We developed gradient boosting ensembles for classification and regression.

### Appendix C.2. Gradient Boosting Machines Algorithm

Gradient boosting refers to a class of ensemble machine learning algorithms that can be used for predictive classification or regression modeling problems. Gradient boosting is also known as gradient tree boosting, stochastic gradient boosting (an extension) and gradient boosting machines, or GBM for short. Ensembles are built from models of decision trees. Trees are added one at a time to the ensemble and fit to correct prediction errors made by previous models. This is an ensemble machine learning model called boosting. Models are fitted using any arbitrary differentiable loss function and any gradient descent optimization algorithm, hence the name of the technique, “gradient boosting”, because the loss gradient is minimized as the model is fitted, just like in a neural network. Naive gradient boosting is a greedy algorithm and can quickly overfit the training dataset. It can benefit from regularization methods that penalize various parts of the algorithm and generally improve the performance of the algorithm by reducing overfitting.

There are three basic types of gradient boosting improvement that can increase its performance:

- Tree constraints: such as the depth of the trees and the number of trees used in the ensemble.
- Weighted updates: such as a learning rate used to limit how much each tree contributes to the ensemble.
- Random sampling: such as fitting trees to random subsets of features and samples. The use of random sampling often leads to a change in the name of the algorithm in “stochastic gradient boosting”.

Gradient boosting is an effective machine learning algorithm and is often the main or one of the main algorithms used to win machine learning competitions (such as Kaggle) [41] on tabular and similar datasets. Next, we described how we fitted GBM models in Python for the specific case of our databases.

### Appendix C.3. Interface (API) Scikit-Learn Gradient Boosting (API)

Boosting gradient sets can be implemented from scratch. However, the machine learning scikit-learn library in Python offers an implementation of gradient boosting ensembles for machine learning. The algorithm is available in a modern version of the library. First, we confirmed that we are using a modern version of the library by running the following script (in Google colab) [42]:

# check scikit-learn version
import sklearn
print(sklearn.__version__)
OUT
0.22.2.post1

Gradient boosting is provided through the GradientBoostingRegressor and GradientBoostingClassifier classes. Both models work the same way and have the same arguments that influence how decision trees are created and added to the ensemble. Random character is used in the construction of the model. This means that, each time the algorithm is run on the same data, it will produce a slightly different model. When using machine learning algorithms that have a stochastic learning algorithm, it is good practice to evaluate them by calculating their performance in several rounds or cross-validation repetitions. When fitting a final model, it is desirable to either increase the number of trees until the variance of the model is reduced during repeated evaluations, or to fit several final models and mediate their predictions.

Next, we will describe how to develop a gradient boosting set for classification.

## Appendix D

# evaluate gradient boosting algorithm for classification
from numpy import mean
from numpy import std
from sklearn.datasets import make_classification
from sklearn.model_selection import cross_val_score
from sklearn.model_selection import RepeatedStratifiedKFold
from sklearn.ensemble import GradientBoostingClassifier

# define the model
model = GradientBoostingClassifier()
# define the evaluation method
cv = RepeaedStratifiedKFold(n_splits = 10, n_repeats = 3, random_state = 1)
# evaluate the model on the dataset
n_scores = cross_val_score(model, X, y, scoring = ‘accuracy’, cv = cv, n_jobs = −1)
# report performance
print(‘Mean Accuracy: %.3f (%.3f)’ % (mean(n_scores), std(n_scores)))

## Appendix E

# example of grid searching key hyperparameters for gradient boosting on a classification dataset
from sklearn.datasets import make_classification
from sklearn.model_selection import RepeatedStratifiedKFold
from sklearn.model_selection import GridSearchCV
from sklearn.ensemble import GradientBoostingClassifier

# define the model with default hyperparameters
model = GradientBoostingClassifier()
# define the grid of values to search
grid = dict()
grid[‘n_estimators’] = [10, 50, 100, 500]
grid[‘learning_rate’] = [0.0001, 0.001, 0.01, 0.1, 1.0]
grid[‘subsample’] = [0.5, 0.7, 1.0]
grid[‘max_depth’] = [3, 7, 9]
# define the evaluation procedure
cv = RepeatedStratifiedKFold(n_splits = 10, n_repeats = 3, random_state = 1)
# define the grid search procedure
grid_search = GridSearchCV(estimator = model, param_grid = grid, n_jobs = −1, cv = cv, scoring = ‘accuracy’)
# execute the grid search
grid_result = grid_search.fit(X, Y)
# summarize the best score and configuration
print(“Best: %f using %s” % (grid_result.best_score_, grid_result.best_params_))

# summarize all scores that were evaluated
means = grid_result.cv_results_[‘mean_test_score’]
stds = grid_result.cv_results_[‘std_test_score’]
params = grid_result.cv_results_[‘params’]
for mean, stdev, param in zip(means, stds, params):
print(“%f (%f) with: %r” % (mean, stdev, param))

## Appendix F

# make predictions using gradient boosting for classification
from sklearn.datasets import make_classification
from sklearn.ensemble import GradientBoostingClassifier
# define the model
model = GradientBoostingClassifier()
# fit the model on the whole dataset
model.fit(X, y)
# make a single prediction
row = [13, 15, 23, 24, 21, 14, 20, 156, 11.62, 23.79, 24, 25.2]
yhat = model.predict([row])
# summarize prediction
print(‘Predicted Class: %d’ % yhat[0])

## Appendix G

# feature selection with univariate statistical tests
from pandas import read_csv
from numpy import set_printoptions
from sklearn.feature_selection import SelectKBest
from sklearn.feature_selection import f_classif

# feature extraction
test = SelectKBest(score_func = f_classif, k = 4)
fit = test.fit(X, Y)
# summarize scores
set_printoptions(precision = 3)
print(fit.scores_)
features = fit.transform(X)
# summarize selected features
print(features[0:5,:])

We can see the scores for each attribute and the 4 chosen features (the ones with the highest scores); specifically, the features with indices 0 (IPQtimeline, 2.201e+00), 3 (IPQperscontrol, 1.178e+00), 8 (hfsub, 5.545e-01) and 11 (famsub, 4.749e-01).

## Appendix H

# feature extraction with RFE
from pandas import read_csv
from sklearn.feature_selection import RFE
from sklearn.linear_model import LogisticRegression

# feature extraction
model = LogisticRegression(solver = ‘lbfgs’)
rfe = RFE(model, 3)
fit = rfe.fit(X, Y)
print(“Num Features: %d” % fit.n_features_)
print(“Selected Features: %s” % fit.support_)
print(“Feature Ranking: %s” % fit.ranking_)

RFE chose the first 3 features as: IPQtimeline, hfsub, pspsub.

These are marked True in *support*_array and marked with option “1” in *ranking*_array.

OUT

Num Features: 3

Selected Features: [ True False False False False False False False True False True False]

Feature Ranking: [ 1 3 7 2 6 4 8 10 1 9 1 5]

## Appendix I

# feature extraction with PCA
import numpy
from pandas import read_csv
from sklearn.decomposition import PCA
# feature extraction
pca = PCA(n_components = 3)
fit = pca.fit(X)
# summarize components
print(“Explained Variance: %s” % fit.explained_variance_ratio_)
print(fit.components_)

## Appendix J

# feature importance with extra trees classifier
from pandas import read_csv
from sklearn.ensemble import ExtraTreesClassifier

# feature extraction
model = ExtraTreesClassifier(n_estimators = 10)
model.fit(X, Y)
print(model.feature_importances_)

We were given an importance score for each attribute; the higher the score, the more important the attribute. The scores suggest the importance of IPQtimeline (0.093), IPQtimecycle (0.115), famsub (0.105).
